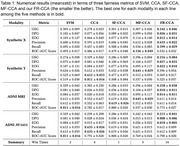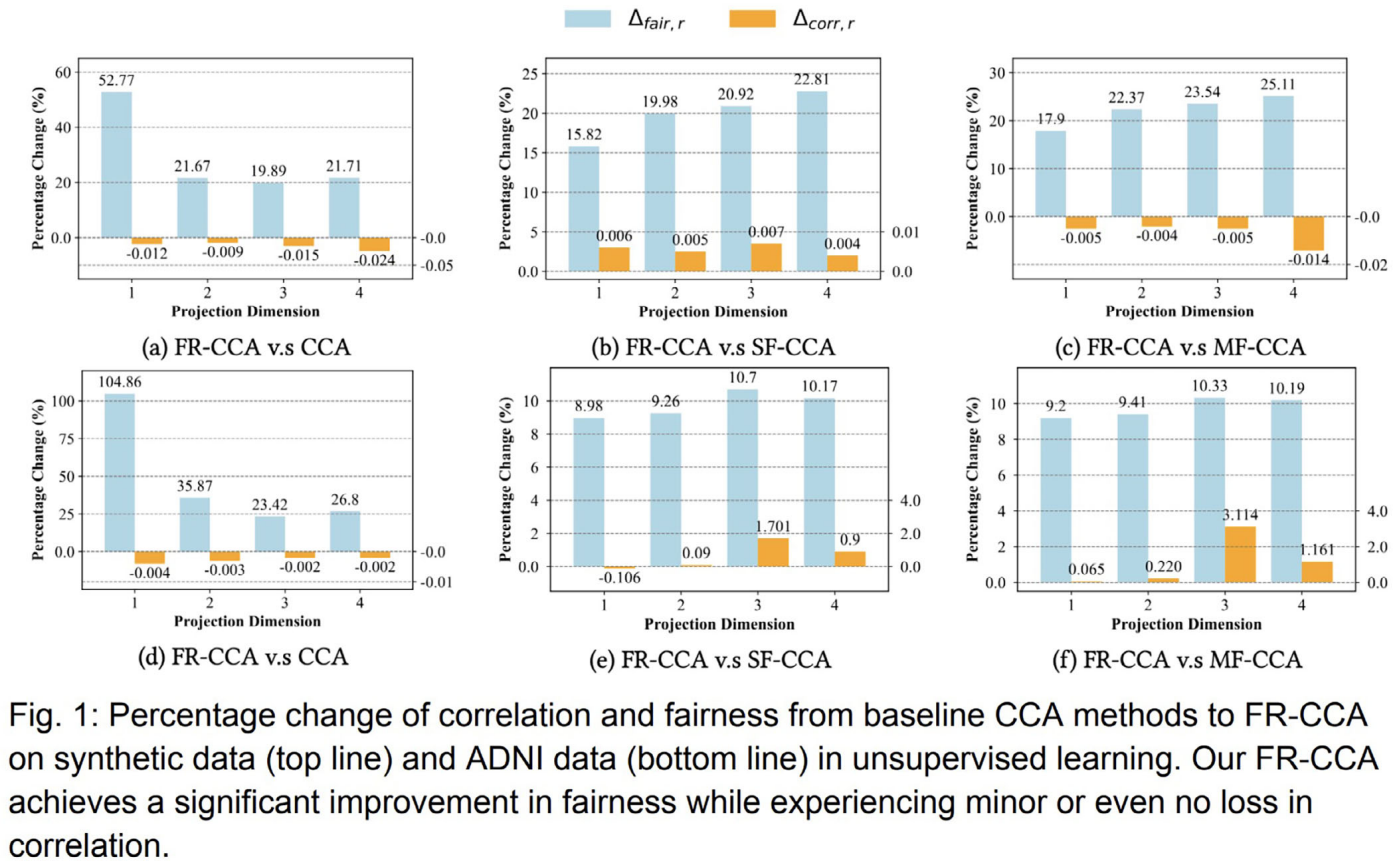# FR‐CCA: Fair Multimodal Representation Learning for Equitable Alzheimer's Disease Diagnosis

**DOI:** 10.1002/alz70855_107221

**Published:** 2025-12-24

**Authors:** Bojian Hou, Zhanliang Wang, Zhuoping Zhou, Boning Tong, Li Shen

**Affiliations:** ^1^ University of Pennsylvania, Philadelphia, PA, USA; ^2^ Department of Biostatistics, Epidemiology, & Informatics, University of Pennsylvania, Philadelphia, PA, USA

## Abstract

**Background:**

Alzheimer's disease (AD) diagnosis increasingly relies on multimodal data integration, combining neuroimaging (MRI/PET) and clinical biomarkers to identify latent disease patterns. However, demographic biases in data collection and population heterogeneity can skew learned representations, leading to disparities in diagnostic accuracy across groups. Traditional dimension reduction methods like CCA ignore these biases, risking amplified inequities in clinical decision‐making.

**Method:**

We propose Fair Representation CCA (FR‐CCA), a novel framework that harmonizes fairness and diagnostic utility in AD biomarker analysis. FR‐CCA learns low‐dimensional representations of multimodal data by maximizing cross‐modal correlations while enforcing statistical independence between latent representations and sensitive attributes (age, sex). We validate FR‐CCA on the Alzheimer's Disease Neuroimaging Initiative (ADNI) dataset, benchmarking against conventional CCA and state‐of‐the‐art fair variants.

**Result:**

FR‐CCA achieves superior fairness‐performance trade‐offs compared to baselines. On synthetic data, fairness improves by 21–52% over CCA with only 1–3% decrease in cross‐modal correlation. For ADNI data, fairness improvements reach 26–105% with negligible correlation loss (<2%). FR‐CCA significantly reduces demographic parity gaps (62–76%) and equality of opportunity gaps (81–88%) while maintaining competitive classification performance. On ADNI data, FR‐CCA reduces sex‐related bias in MRI and AV1451 PET modalities by 84–90% (demographic parity gap: 0.010±0.011 vs. 0.104±0.057 for MF‐CCA in MRI) with minimal impact on diagnostic accuracy (ROC‐AUC: 0.773±0.027 vs. 0.782±0.017 for CCA in MRI). Overall, FR‐CCA achieves the highest performance across 14/22 metrics, validating its robustness.

**Conclusion:**

FR‐CCA provides a clinically actionable solution for equitable AD diagnosis by addressing biases in multimodal data integration. Its ability to balance fairness and accuracy ensures diagnostic models generalize robustly across diverse populations, addressing critical gaps in healthcare equity and offering a scalable framework for other high‐stakes medical applications.